# Intermittent Hypoxia Increases the Severity of Bleomycin-Induced Lung Injury in Mice

**DOI:** 10.1155/2018/1240192

**Published:** 2018-03-14

**Authors:** Thomas Gille, Morgane Didier, Cécile Rotenberg, Eva Delbrel, Dominique Marchant, Angela Sutton, Nicolas Dard, Liasmine Haine, Nicolas Voituron, Jean-François Bernaudin, Dominique Valeyre, Hilario Nunes, Valérie Besnard, Emilie Boncoeur, Carole Planès

**Affiliations:** ^1^Laboratoire Hypoxie & Poumon (EA 2363), Université Paris 13, Sorbonne Paris Cité, 93017 Bobigny, France; ^2^Hôpital Avicenne (AP-HP), 93009 Bobigny, France; ^3^AP-HP, Hôpital Jean Verdier, 93140 Bondy, France; ^4^UPMC, Sorbonne Université, 75013 Paris, France

## Abstract

**Background:**

Severe obstructive sleep apnea (OSA) with chronic intermittent hypoxia (IH) is common in idiopathic pulmonary fibrosis (IPF). Here, we evaluated the impact of IH on bleomycin- (BLM-) induced pulmonary fibrosis in mice.

**Methods:**

C57BL/6J mice received intratracheal BLM or saline and were exposed to IH (40 cycles/hour; FiO_2_ nadir: 6%; 8 hours/day) or intermittent air (IA). In the four experimental groups, we evaluated (i) survival; (ii) alveolar inflammation, pulmonary edema, lung oxidative stress, and antioxidant enzymes; (iii) lung cell apoptosis; and (iv) pulmonary fibrosis.

**Results:**

Survival at day 21 was lower in the BLM-IH group (*p* < 0.05). Pulmonary fibrosis was more severe at day 21 in BLM-IH mice, as assessed by lung collagen content (*p* = 0.02) and histology. At day 4, BLM-IH mice developed a more severe neutrophilic alveolitis, (*p* < 0.001). Lung oxidative stress was observed, and superoxide dismutase and glutathione peroxidase expression was decreased in BLM-IH mice (*p* < 0.05 versus BLM-IA group). At day 8, pulmonary edema was observed and lung cell apoptosis was increased in the BLM-IH group.

**Conclusion:**

These results show that exposure to chronic IH increases mortality, lung inflammation, and lung fibrosis in BLM-treated mice. This study raises the question of the worsening impact of severe OSA in IPF patients.

## 1. Introduction

Several studies recently showed that obstructive sleep apnea syndrome (OSA) was highly established in incident and prevalent cases of idiopathic pulmonary fibrosis (IPF) [1–4]. We reported in a prospective study that the prevalence of moderate-to-severe and of severe OSA was 62% and 40%, respectively, in patients with newly diagnosed IPF, suggesting that OSA precedes (or occurs at the same time as) the onset of IPF [1]. Indeed in the IPF population, severe OSA was strongly associated with the presence of cardiovascular diseases (particularly ischemic heart disease) and with increased systemic oxidative stress and IPF biomarkers. IPF, the most common form of idiopathic interstitial pneumonias, is a devastating lung disease with a median survival of ~3 years from the time of diagnosis, for which medical treatments show little efficacy [5, 6]. IPF generally affects subjects over 60 and is characterized by the accumulation of extracellular matrix in the distal regions of the lungs, leading to impairment of alveolar gas exchange and progressive loss of pulmonary function. The pathophysiology of IPF is not fully understood, but it could be due to aberrant repair following repetitive microinjuries of the alveolar epithelium, including chronic silent microaspiration, cigarette smoke, and chronic viral infections for example [5, 7]. Oxidative stress is observed in the lung of IPF patients and most likely plays an important role in the pathophysiology of the disease [8, 9].

Although the prognosis of IPF is generally bad, there is a great heterogeneity in the natural course of the disease, which could be explained by the presence of associated comorbidities [6]. In patients with IPF, the potential influence of OSA on the natural course of lung fibrosis has never been evaluated using robust methodology. Here, we hypothesized that moderate-to-severe OSA, by increasing lung oxidative stress and inflammation through chronic nocturnal intermittent hypoxia (IH) [10–12], could lead to repetitive alveolar microinjuries promoting the development/progression of pulmonary fibrosis [13]. To test this hypothesis, we set up a double-hit experimental model in which mice were first treated with bleomycin (BLM) to induce lung fibrosis and then exposed to chronic IH or intermittent air for 3 weeks. The objectives of the present study were to evaluate in this model the effects of chronic IH on animal survival and the severity of lung inflammation and of lung fibrosis and to investigate the mechanism(s) involved. Our results show that exposure to chronic IH mimicking severe OSA increased animal mortality and worsened lung inflammation and lung fibrosis in BLM-treated mice.

## 2. Methods

### 2.1. Animals

Male C57BL/6J mice were purchased from Janvier Labs (Le Genest-Saint-Isle, France). All experiments were approved by the institutional reviewing board on animal experimentation and accorded with animal welfare guidelines (Ministère Français de la Pêche et de l'Agriculture, APAFIS number 1258-2015072312175063). Animals were housed in standard cages and light conditions and fed standard diet with ad libitum access to drinking water in our University Animal Facility (Agreement number A 9300801). Experiments were performed on 2-3-month-old mice, with investigators blinded to experimental condition for all comparative measurements. The total number of mice used in this study was 100.

### 2.2. Model of Pulmonary Fibrosis and Chronic Intermittent Hypoxia

Pulmonary fibrosis was induced by a single intratracheal instillation of bleomycin (BLM) (Bellon, Paris, France) (3.5 unit/g body weight in 100 *μ*l saline) [14, 15]. In preliminary experiments, such a dose of BLM induced significant pulmonary fibrosis on day 21 as assessed by lung histology and collagen content determination with an acceptable mortality rate (~25% on day 21). Exposure to chronic intermittent hypoxia (IH) was achieved in customized Plexiglas chambers using an automated nitrogen/air delivery profile system (O_2_ Sense Gas Driver Vivo, Adelbio, Aubière, France) modulating flow rates and adjusting the balance of inspired nitrogen and room air enriched with oxygen (O_2_). The fraction of inspired oxygen (FIO_2_) within the chamber declined from 21% to 6%, resulting in arterial oxyhemoglobin saturation nadir of 60% to 65% for <5 seconds in mice. The nadir in FIO_2_ within the chamber during each cycle was followed by restoration of oxygen tension to 21% by flushing the chamber with room air enriched with O_2_. Control animals exposed to chronic intermittent air (IA) were placed in a similar Plexiglas chamber in the same room and exposed to alternating periods of room air using an identical protocol of gas flows as the IH protocol. Exposures were conducted for 40 cycles/h (duration of each cycle: 90 s), 8 h/day (09:00–17:00, during the rodent nocturnal cycle), and 7 days/week for up to 3 weeks.

### 2.3. Experimental Design

**Figure 1:**
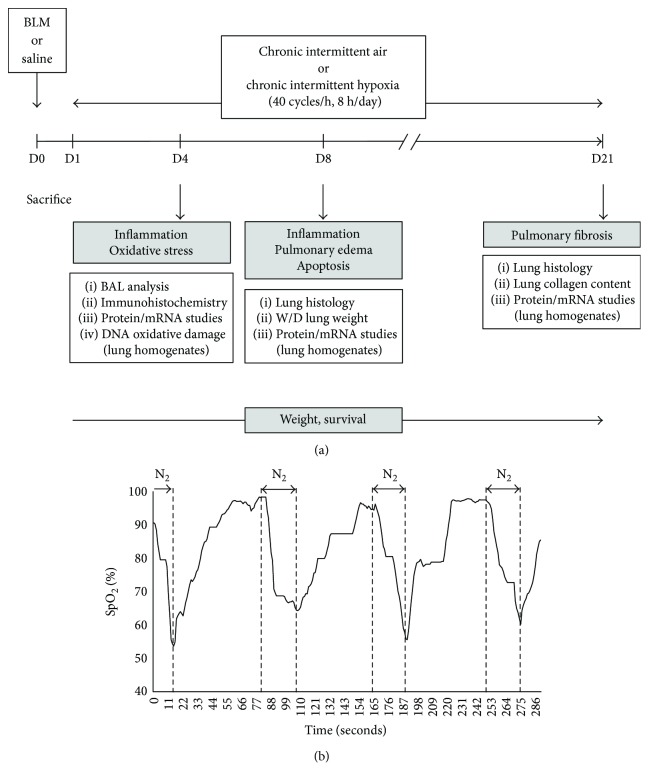
Exposure to intermittent hypoxia in a murine model of lung fibrosis. (a) Experimental design. (b) Representative trace of oxygen saturation measured by pulse oximetry (SpO_2_) in a mouse exposed to intermittent hypoxia.

The experimental design is shown in Figure 1. On day 0, anesthetized mice were given intratracheal BLM or saline and allowed to recover for 24 h. On day 1, mice were exposed to either IH or IA as described above for 4, 8, or 21 days. Therefore, 4 experimental groups were studied: group *saline + chronic intermittent air* (Saline-IA), group *saline + chronic intermittent hypoxia* (Saline-IH), group *bleomycin + chronic intermittent air* (BLM-IA), and group *bleomycin + chronic intermittent hypoxia* (BLM-IH). Animal viability was checked twice a day, and mice were weighed on alternate days. Mice were sacrificed on day 4, day 8, or day 21. Briefly, mice were euthanized with intraperitoneal injection of pentobarbital (250 mg/kg) followed by exsanguination. The trachea was cannulated before a thoracotomy was performed, and a catheter was inserted in the pulmonary artery to perform lung vascular lavage. Lungs were processed for further experiments as described below.

### 2.4. Lung Histology

Lung histology was performed as previously described [16]. Lungs were inflated with 4% paraformaldehyde at a pressure of 20 cm H_2_O before the trachea was tied. Heart and lungs were removed *en bloc* and placed in 20 ml 4% paraformaldehyde overnight. The lungs were embedded in paraffin. Sections were cut at 4 *μ*m thickness and stained with haematoxylin and eosin.

### 2.5. Bronchoalveolar Lavage

One ml of cold saline was injected into the lungs through the tracheal cannula and flushed back and forth three times. The bronchoalveolar lavage (BAL) fluid was centrifuged, and the supernatant was immediately frozen at −80°C. The cell pellet was further processed to obtain total cell count and cytological formula.

### 2.6. Determination of Lung Wet/Dry Weight Ratio

Lung water content (including intravascular water, interstitial, and alveolar water) was determined by the lung wet/dry weight ratio as previously described [17]. Briefly, mice were killed as described above except that lung vascular lavage was not performed. The right lower lobe was removed, weighed, then placed in an incubator at 80°C for 24 h for desiccation and weighed again to calculate the wet/dry lung weight ratio.

### 2.7. Immunohistochemistry

Immunolocalization of 3-nitrotyrosine was performed on 4 *μ*m paraffin-embedded lung sections from the 4 experimental groups (4-5 per group). Sections were deparaffinized and rehydrated, and epitopes were recovered using 0.01 M sodium citrate buffer at pH 6. Then, tissue sections were incubated overnight at 4°C with primary antibody (1 : 250 for anti 3-nitrotyrosine (Abcam number ab61392, France). The LSAB2/DAB kit (Dako/Agilent Technologies, France) was used to detect bound antibodies, according to the manufacturer's instructions, and tissue sections were counterstained with haematoxylin.

### 2.8. TUNEL Experiments

The detection and quantification of apoptosis by TUNEL staining were performed on paraffin-embedded lung sections from the 4 experimental groups, using an In Situ Cell Death Detection kit from the ROCHE company (number 11684795910). Briefly, tissue sections were dewaxed, rehydrated, and permeabilized using proteinase K. A positive control was performed using DNase I. The labelling protocol was performed following the manufacturer's instructions. Slides were mounted using Vectashield Antifade Mounting Medium with DAPI (Vector number H-1200, United Kingdom).

### 2.9. Morphometry

Counting of nitrotyrosine-positive cells was performed on lung sections of four to five mice for each experimental group, and results were normalized per lung area. The overall proportion of TUNEL-positive cells was determined by dividing the number of TUNEL-positive cells (fluorescein-positive cells) by the total number of cells contained within the field (DAPI stained cells) and then multiplying by 100. For both counting, five fields per section were analyzed to gather the data. The *x-* and *y*-coordinates for each field measured were selected by a random number generator. Double-blind counting was performed for both nitrotyrosine immunohistostaining and TUNEL assay.

### 2.10. ELISA

Myeloperoxidase (MPO) concentration was assessed in 40 *μ*g lung homogenates by ELISA according to the manufacturer's instructions (DuoSet DY3667, Bio-Techne). DNA oxidative damage was assessed in 40 *μ*g lung homogenates and BAL fluid by determination of 8-hydroxy-deoxyguanosine (8-OH-DG) levels using an ELISA kit (Oxiselect™, Cell Biolabs Inc., San Diego, CA).

### 2.11. Western Blot Analysis

Lungs were removed and immediately homogenized for 3 min in ice-cold lysis RIPA buffer (pH 8) containing 20 mM Tris, 150 mM NaCl, 1% Triton X-100, 0.1% SDS, 0.5% deoxycholate, and protease inhibitors. The lysate was centrifuged (15.000 rpm, 10 min, 4°C), and supernatants were aliquoted and immediately frozen before use. For Western blotting, samples of protein extracts (40 or 100 *μ*g/lane) were separated by SDS-PAGE and transferred onto nitrocellulose membrane (GE Healthcare). Membranes were blocked in TBST (150 mM NaCl, 10 mM Tris, pH 7.4, 0.1% Tween 20) containing 5% (*w*/*v*) dry milk powder and then incubated with primary antibodies (diluted in blocking solution) overnight at 4°C. Following washes with TBST, blots were incubated for 1 hr at room temperature with secondary antibodies. Immunoreactive bands were revealed with the ECL Luminata kit (Millipore) or West kit (Thermo Scientific), visualized on an image capture system (ChemiDoc MP, Bio-Rad), and quantified with Image Lab software (Bio-Rad). Protein levels were normalized to respective *β*-actin control. Primary antibodies used in the study were rabbit polyclonal anti superoxide dismutase 1 (SOD1) (Enzo Life Sciences, ADI-SOD 100; dilution: 1/5000), polyclonal rabbit anti superoxide dismutase 2 (SOD2) (Enzo Life Sciences, ADI-SOD 100; dilution: 1/5000), rabbit polyclonal anti-catalase (Calbiochem, 219010; dilution: 1/5000), rabbit polyclonal anti glutathione peroxidase (GPX) (Abcam, ab59546; dilution 1/5000), rabbit polyclonal anti-cleaved PARP (Poly-ADP-ribose polymerase) (Cell Signalling; dilution:1/1000), and mouse monoclonal anti *β*-actin (Santa Cruz, SC 47778; dilution: 1/1000).

### 2.12. Reverse Transcription and Real-Time (RT) PCR Analysis

Total cellular RNAs from lung homogenates were extracted using the RNeasy kit (QIAGEN S.A., Courtaboeuf, France) following the manufacturer's instructions. RNAs were quantified using a BioSpec-nano (Shimadzu, Noisiel, France) at 260 nm. Single-strand cDNAs were synthesized from 0.5 *μ*g of total RNA using Maxima first strand cDNA synthesis kit composed by a mixture of oligo (dT) and random hexamer primers according to the manufacturer's instructions (Fisher Scientific, Illkirch, France). Resulting cDNA samples were amplified by quantitative polymerase chain reaction (PCR) with Absolute qPCR SYBR Green ROX mix (Fisher Scientific, Illkirch, France) on StepOne system qPCR (Applied Biosystems, Life Technologies, France). Cycle threshold values were normalized to amplification of *Beta 2 microglobulin (B2m)*. For each transcript, the expression levels were calculated using the 2–∆∆CT method, as detailed by the manufacturer. Primer sequences used for quantitative real-time PCR are listed in Table 1.

### 2.13. Lung Collagen Content

Soluble lung collagen content was measured in the frozen unlavaged right lung with the Sircol assay (Biocolor Ltd., Belfast, UK) according to the manufacturer's instructions. Results were expressed in *μ*g/lobe.

### 2.14. Statistical Analysis

Results are presented as means ± SE. Differences between groups were evaluated with one-way variance analyses (ANOVA), and, when allowed by the *F* value, results were compared by the modified least significant difference (Fisher's PLSD). Survival was estimated by the method of Kaplan-Meier and compared by the log-rank test. *p* < 0.05 was considered significant. Analyses were carried out using StatView® software (SAS Institute Inc., Cary, NC, USA) and Prism® software (GraphPad Software Inc., La Jolla, CA, USA).

## 3. Results

### 3.1. Exposure to Intermittent Hypoxia Reduces Survival in Bleomycin-Treated Mice

**Figure 2:**
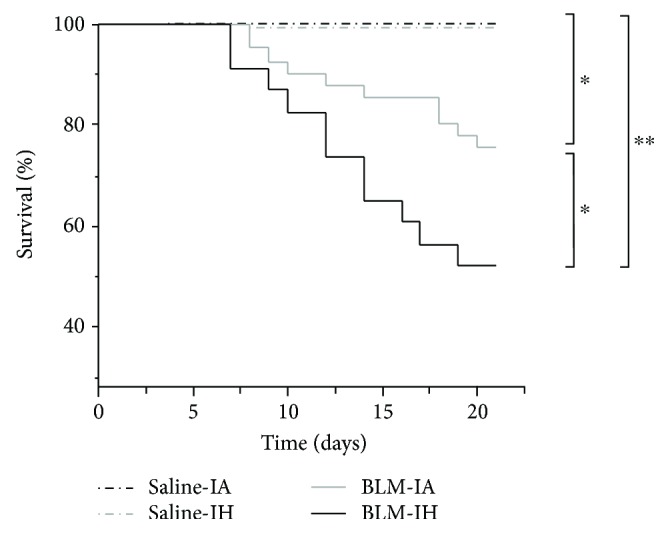
Survival is reduced in response to intermittent hypoxia in a murine model of lung fibrosis. Kaplan-Meier plot of mice survival after bleomycin exposure in normoxia and intermittent hypoxia (IH). Eight-week-old mice received an intratracheal instillation of bleomycin or saline (NaCl) at day 0. Survival of mice (*n* = 23–41) after bleomycin was significantly decreased compared with controls (*n* = 14–16). Exposure to IH worsened mice survival. *p* < 0.0001 by Log-rank test. ^∗^*p* < 0.05 versus Saline-IA; ^∗∗^*p* < 0.01 versus Saline-IA.

Global comparisons revealed significant differences in survival between groups of mice treated with saline or BLM at day 0 and exposed to IA or IH from day 1 to day 21, as shown in Figure 2. In mice treated with saline and exposed to either AI or HI, the survival rate at day 21 was 100%. Treatment with BLM induced mortality after day 8 in mice exposed to either IA or IH. Indeed, the survival over 21 days was significantly lower in mice treated with BLM exposed to IH than in mice treated with BLM exposed to IA (52% versus 76%, *p* = 0.04). Gross examination of lungs from all deceased mice revealed the presence of large congestive hemorrhagic areas.

### 3.2. Exposure to Intermittent Hypoxia Increases Lung Inflammation and Pulmonary Edema in Bleomycin-Treated Mice

**Figure 3:**
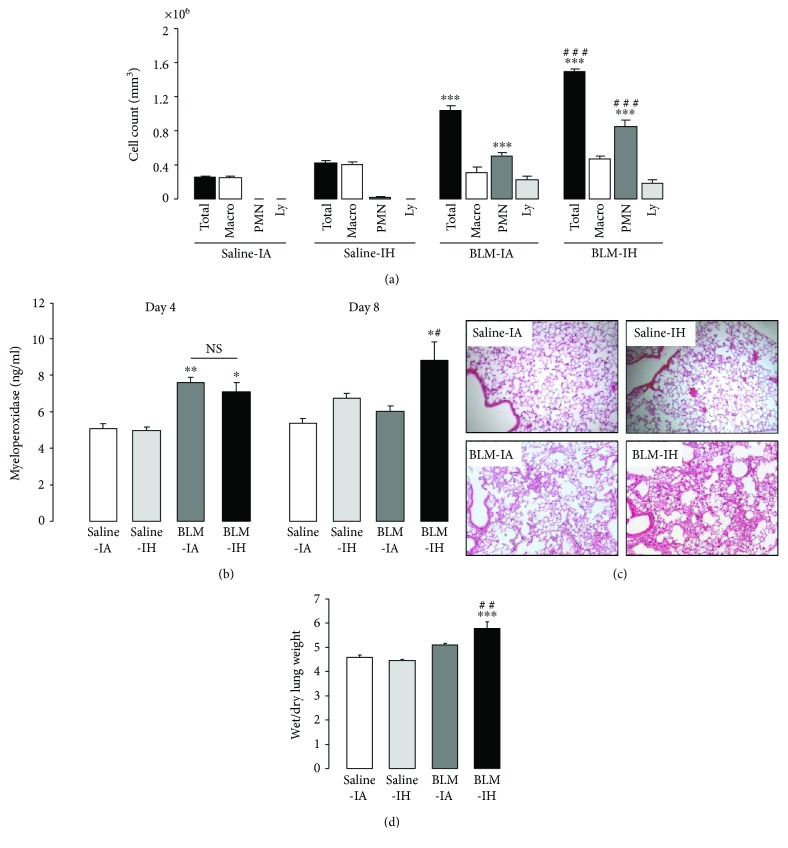
Intermittent hypoxia increases lung inflammation and pulmonary edema in bleomycin-treated mice. (a) Total cell count was determined in BAL from mice at day 4 in the 4 experimental groups. Changes in BAL cell population (Macro: macrophages; PMN: polymorphonuclear cells; Ly: lymphocytes) were determined in the 4 experimental groups. Data represent means ± SE, *n* = 6 mice per group. (b) Myeloperoxidase (MPO) concentration was assessed by ELISA in lung homogenate of mice at day 4 and day 8 in the 4 experimental groups. Data represent means ± SE, *n* = 4 mice per group. (c) Lung histology of mice at day 8. Lung sections were prepared at day 8 in the 4 experimental groups and stained with haematoxylin and eosin to assess lung morphology. (d) Lung wet/dry weight ratios for all experimental groups at day 8. Data represent means ± SE, *n* = 5 mice per group. ^∗^*p* < 0.01 versus Saline-IA; ^∗∗^*p* < 0.01 versus Saline-IA; ^∗∗∗^*p* < 0.001 versus Saline-IA; ^#^*p* < 0.05 versus BLM-IA; ^##^*p* < 0.01 versus BLM-IA; ^###^*p* < 0.001 versus BLM-IA.

Total cell counts in BAL at day 4 were significantly increased in mice treated with BLM (whether exposed to IA or IH), as compared with mice treated with saline (Figure 3(a)). This was associated with an increase in the number of polymorphonuclear cells (PMN). The increase in BAL total cell count and the increase in PMN number were significantly more important in the BLM IH group than in the BLM IA group (*p* < 0.001), indicating that exposure to IH exacerbated the neutrophilic alveolitis induced by BLM treatment. As shown in Figure 3(b), concentration of myeloperoxidase (MPO) in lung homogenates at day 4 was significantly induced in groups of mice treated with BLM, with no difference between mice exposed to IA or IH. On day 8, MPO concentration had returned to normal in mice treated with BLM and IA, but was still significantly increased in mice treated with BLM and IH (*p* < 0.05). Histological analysis of lung tissue at day 8 clearly showed alveolar and interstitial cellular infiltrates in the lungs of BLM-treated mice that appeared with a larger extent in the BLM-IH group than in the BLM-IA group (Figure 3(c)). Also, the wet-to-dry lung weight ratio at day 8 was significantly increased in the BLM-IH group, indicating the presence of pulmonary edema.

### 3.3. Lung Oxidative Stress and Lung Antioxidant Enzymes

**Figure 4:**
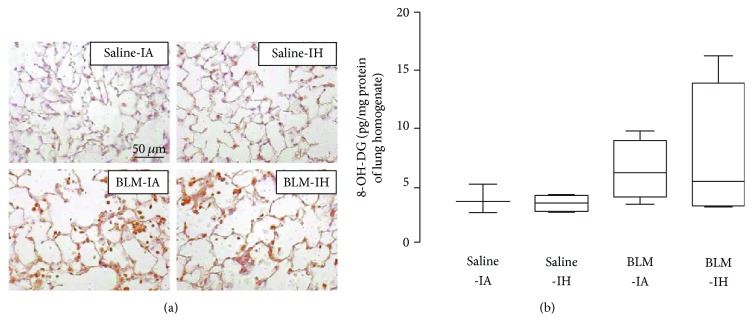
Intermittent hypoxia and bleomycin enhanced lung nitrosative/oxidative stress in mouse lungs. (a) 3-nitrotyrosine presence was assessed by immunostaining on lung sections of mice prepared at day 4 in the 4 experimental groups. The figure is representative of at least 5 individual mice for each group. Magnification ×200. (b) 8-OH-DG concentration was assessed by ELISA in lung homogenate of mice at day 4 in the 4 experimental groups. Data represent means ± SE, *n* = 4-5 mice per group.

**Figure 5:**
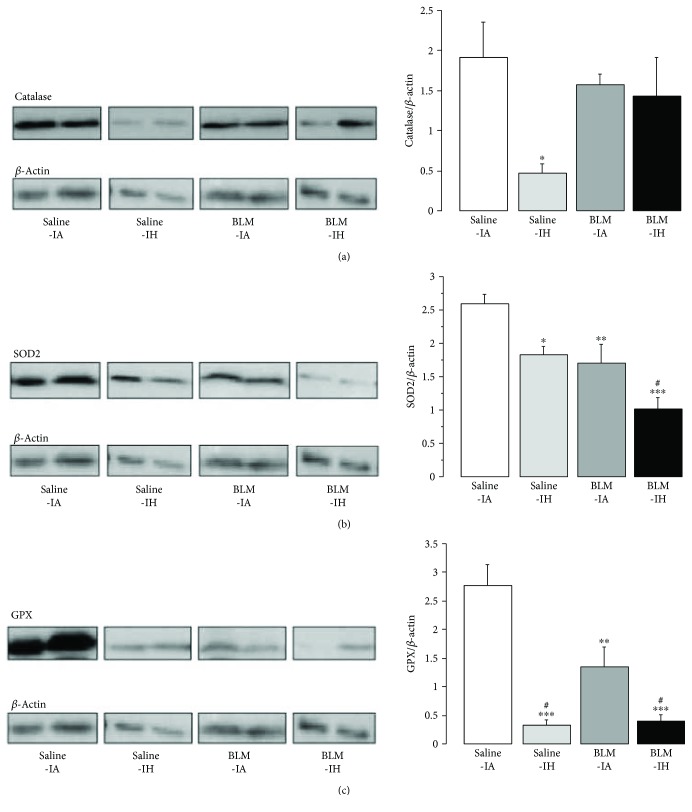
Intermittent hypoxia reduced antioxidant enzymes in mouse lungs. Immunoblotting for catalase (a), SOD2 (b), and GPX (c) was performed on lungs on D4 in the 4 experimental groups. Histograms show quantitative representation of protein levels of *n* = 4-5 mice/group. ^∗^*p* < 0.01 versus Saline-IA; ^∗∗^*p* < 0.01 versus Saline-IA; ^∗∗∗^*p* < 0.001 versus Saline-IA; ^#^*p* < 0.05 versus BLM-IA.

Lung oxidative stress was evaluated at day 4 by 3-nitrotyrosine immunostaining of lung sections (reflecting protein nitrosylation) [18] and by assessment of 8-OH-DG levels [1] in lung homogenates and BAL fluid. Immunostaining of 3-nitrotyrosine was very weak in lung sections from mice treated with saline and either exposed to IA or to IH (*n* = 4-5 per group) (Figure 4(a)). By contrast, staining was observed in all lung sections from mice treated with BLM, with no obvious difference between the BLM-IA group and the BLM-IH group (*n* = 4-5 per group). 3-nitrotyrosine-positive cells were both immune cells and alveolar epithelial cells (Figure 4(a)). As shown in Figure 4(b), there was a trend to an increase in 8-OH-DG levels in lung homogenates from mice treated with BLM (the BLM-IA and BLM-IH groups) as compared with mice treated with saline. However, this increase was not significant, most likely because of the small number of samples and the variability of values in BLM-treated mice. Measurements of 8-OH-DG in BAL fluid samples showed that 8-OH-DG levels were under the detection threshold of the ELISA kit in all mice treated with saline (data not shown). Concerning BLM-treated mice, 8-OH-DG was detected (i.e., values above the detection threshold) in 3 upon 9 BAL fluid samples in the BLM-IA, and in 5 upon 7 samples in the BLM-IH group (*p* = 0.07 by *Chi2*). Next, we assessed protein expression levels of antioxidant enzymes (SOD1, SOD2, catalase, and GPX) by Western blotting in lung homogenates at day 4 (Figure 5). Expression levels of SOD1 were not significantly modified by any experimental condition (data not shown). Expression of catalase in lung homogenates was markedly decreased by 76% in the Saline-IH group as compared with the Saline-IA group (*p* < 0.5), but was not significantly modified in the BLM-IA and BLM-IH groups (Figure 5(a)). Expression levels of SOD2 protein were significantly decreased by 30–35% in the Saline-IH and the BLM-IA groups as compared with the Saline-IA group and were further decreased (−61%) in the BLM-IH group (Figure 5(b)). Expression levels of SOD2 were significantly lower in the BLM-IH group than in the BLM-IA group (*p* < 0.05). Finally, expression levels of GPX were reduced by 50% in the BLM-AI group as compared with the Saline-AI group (*p* < 0.01) and were further reduced by more than 85% in the Saline-IH and the BLM-IH groups (*p* < 0.001 as compared with the Saline-IA group and *p* < 0.05 as compared with the BLM-IA group) (Figure 5(c)).

### 3.4. Exposure to Intermittent Hypoxia Increases Lung Cell Apoptosis in Bleomycin-Treated Mice

**Figure 6:**
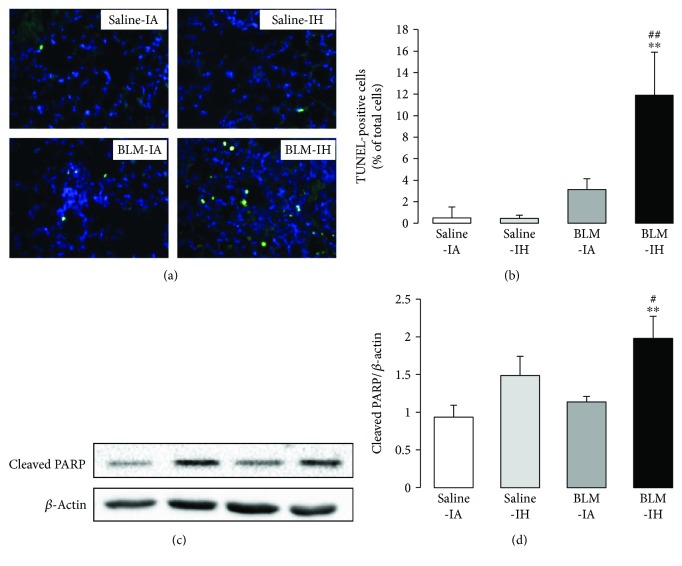
Intermittent hypoxia increased apoptosis in mouse lungs. (a) Apoptosis was determined by TUNEL assay on lung sections of mice prepared at day 8 in the 4 experimental groups. Magnification ×200. (b) Fraction of TUNEL-positive cells (green) on lung sections was determined relative to DAPI-positive cells (blue). The figure is representative of at least 5 individual mice for each group. (c) Immunoblotting for cleaved PARP was performed on lungs on day 8 in the 4 experimental groups. (d) Histograms show quantitative representation of protein levels of *n* = 4-5 mice/group. ^∗∗^*p* < 0.01 versus Saline-IA; ^#^*p* < 0.05 versus BLM-IA; ^##^*p* < 0.01 versus BLM-IA.

Lung cell apoptosis was evaluated at day 8 by TUNEL staining on paraffin-embedded lung sections and by quantification of cleaved-PARP protein expression levels reflecting early apoptosis [19] by Western blot in lung homogenates (Figure 6). The number of TUNEL-positive cells was very low in Saline-IA- and Saline-IF-treated lungs, tended to increase in BLM-IA lungs (not significant), and was significantly augmented in BLM-IH lungs (Figures 6(a) and 6(b)). TUNEL-positive cells were mostly alveolar epithelial type II cells, and to a lesser degree intra-alveolar inflammatory cells. Consistent with this result, the protein expression level of cleaved-PARP was significantly increased in lung homogenates from BLM-IH-treated mice as compared with other groups (Figures 6(c) and 6(d)).

### 3.5. Exposure to Intermittent Hypoxia Worsens Lung Fibrosis in Bleomycin-Treated Mice

**Figure 7:**
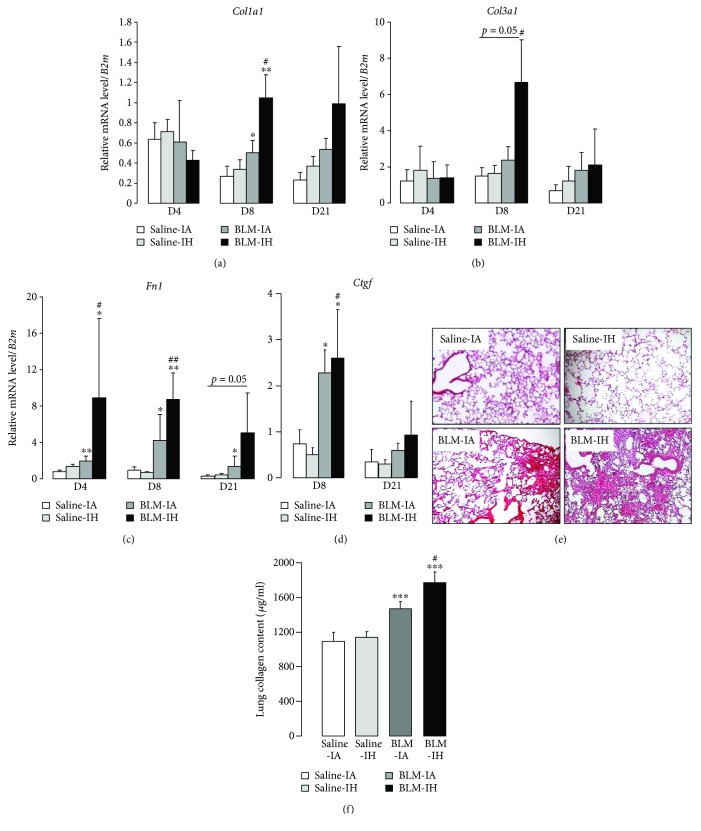
Lung fibrosis is increased in response to intermittent hypoxia. Quantitative RT-PCR was performed to estimate (a) *Col1a1*, (b) *Col3a1*, (c) *Fn1*, and (d) *Ctgf* mRNA levels in whole lung homogenate from mice in the 4 experimental groups and normalized to *B2m* mRNA. Results are expressed as means ± SE of 5 animals per group. (e) Lung histology of mice at day 21. Lung sections were prepared at day 21 in the 4 experimental groups and stained with haematoxylin and eosin to assess lung morphology. (f) Soluble collagen content was assessed by Sircol assay in lung homogenates in saline-treated mice (*n* = 5–10 mice per group) compared with BLM-treated mice (*n* = 14–18 mice per group). ^∗^*p* < 0.01 versus Saline-IA; ^∗∗^*p* < 0.01 versus Saline-IA; ^∗∗∗^*p* < 0.001 versus Saline-IA; ^#^*p* < 0.05 versus BLM-IA; ^##^*p* < 0.01 versus BLM-IA.

First, levels of mRNA transcripts coding for extracellular matrix proteins (*Col1a1*, *Col3a1*, and *Fn1*) and profibrotic factors (*Tgfb1* and *Ctgf*) were evaluated in lung homogenates by qRT-PCR at day 4, day 8, and day 21 (Figures 7(a)–7(c)). Exposure to IH per se did not modify mRNA expression levels, as compared with IA. Treatment with BLM + IA induced a significant increase in *Fn1* mRNA levels at day 4, day 8, and day 21 (as compared with saline) (Figure 7(c)), and in *Col1a1* mRNA levels at day 8 (Figure 7(a)). Treatment with BLM + IH further increased *Col1a1* and *Fn1* mRNA transcript levels at day 8 (Figures 7(a) and 7(b)), and also increased *Col3a1* mRNA transcript levels at day 8 (*p* = 0.05) (Figure 7(b)). Levels of *Tgfb1* mRNA transcripts were not modified in any experimental condition (data not shown). Expression levels of *Ctgf* mRNAs were significantly increased at day 8 in mouse lungs treated with BLM, with no significant difference between the BLM-IA and the BLM-IH group, and returned to normal at day 21 (Figure 7(d)). In addition, histological examination at day 21 revealed a patchy distribution of fibrotic areas in the lungs from mice treated with BLM (Figure 7(d)). Lung fibrosis appeared more severe with a larger extent of fibrotic areas in the BLM IH group than in the BLM IA group (Figure 7(e), lower panels). Finally, quantification of collagen content in lung homogenates by Sircol assay at day 21 (Figure 7(e)) showed that collagen content was increased by 35% and by 63% in the BLM IA group and in the BLM IH group, respectively, as compared with Saline IA group (*p* < 0.001). Lung collagen content was significantly higher in the BLM IH group than in the BLM IA group (1772 ± 124 versus 1470 ± 82 *μ*g/ml, *p* < 0.05). Taken together, these data indicate that exposure to IH worsened lung fibrosis in BLM-treated mice.

## 4. Discussion

The high prevalence of moderate-to-severe OSA in prevalent and incident cases of IPF raises the question whether OSA could modulate the course of pulmonary fibrosis [1–3]. In the present study, we evaluated the effect of chronic IH, a typical feature of severe OSA [10, 11, 20], on the severity of lung fibrosis induced by BLM in mice. Our main findings provide evidence that exposure to chronic IH worsened BLM-induced lung injury inasmuch as IH: (i) doubled mortality after 21 days in BLM-treated mice; (ii) increased pulmonary inflammation as assessed by neutrophilic alveolitis and pulmonary edema during the first week following BLM treatment; (iii) induced lung cell apoptosis; and (iv) increased the severity of BLM-induced pulmonary fibrosis estimated by histological analysis, *collagen* and *fibronectin* mRNA transcript levels, and collagen content in lung homogenates. Indeed, exposure of BLM-treated mice to chronic IH was associated with an imbalance between antioxidant and prooxidant enzymes compromising cellular defense against oxidative stress.

IPF is thought to be the consequence of repetitive microinjuries on the ageing lung, followed by inefficient repair of the injured alveolar epithelium, and uncontrolled activation and proliferation of (myo)fibroblasts [5]. ATII cells, the progenitor cells of alveolar epithelium, instead of proliferating and transdifferentiating to recover the denuded alveolar basal membrane, undergo massive apoptosis or may even transform themselves into fibroblasts through epithelial-mesenchymal transition (EMT) [21, 22]. Some ATII cells also become hyperplastic with abnormal activation and production of profibrotic factors such as TGF*β*1 and CTGF. IPF lungs display increased oxidative stress probably caused by an increase in oxidants associated with extracellular glutathione deficiency [8, 9]. In the present study, experimental lung fibrosis was induced in mice by a single intratracheal instillation of BLM. The BLM model is the best-characterized murine model of pulmonary fibrosis [14, 15]. BLM, a drug with antibiotic/antineoplastic properties originally isolated from *Streptomyces verticillatus*, has a dose-dependent pulmonary toxicity resulting in lung fibrosis in humans. Intratracheal administration of BLM in murine lung induces a direct damage of alveolar epithelial cells (mostly alveolar type I cells), an inflammatory response, and increased epithelial apoptosis, with signs resembling acute lung injury within the first week. In response to this initial injury, lung fibrosis progressively develops to be maximal around days 21–28 and can eventually resolve afterwards. Although the BLM model does not recapitulate all features of the IPF lung, it is the most extensively used murine model to study the pathogenesis of pulmonary fibrosis. In our model, mice were exposed one day after BLM administration to chronic and severe IH (40 cycles/h, 8 h/day, nadir FIO_2_ 6%, for 21 days) to mimic nocturnal hypoxia-reoxygenation episodes encountered during severe OSA, inasmuch as severe OSA may affect as much as 40% of patients with incident IPF [1]. Indeed, previous animal studies have shown that such regimens of chronic and severe IH were able to induce in mice deleterious cardiovascular, metabolic, or neurological effects, whereas less severe regimens (shorter duration, lower cycle frequency, higher FIO_2_ nadir) were not or could even exert some protective effects [10, 11].

In our model, lung inflammation was evidenced within the first week in mice treated with BLM, but not in mice treated with saline and exposed to either IA or IH. Interestingly, lung inflammation was more intense in mice treated with BLM and IH than in those treated with BLM and IA, as indicated by histological analysis, a more severe neutrophilic alveolitis at day 4 and a greater increase in MPO expression in lung homogenates at day 8. Also, pulmonary edema as assessed by wet-to-dry lung weight ratio at day 8 was more severe in the BLM-IH group than in the BLM-IA group. The severity of lung edema in mice treated with BLM and IH could be due to increased air-blood barrier permeability to fluid and proteins leading to alveolar flooding, secondary to the afflux and activation of PMN as described in acute lung injury [23]. In addition, accumulation of pulmonary edema fluid can be the consequence of decreased ability of the lung to clear edema fluid from the alveolar space. Transepithelial vectorial sodium transport from alveoli to interstitium is achieved by alveolar epithelial cells and normally provides the driving force for edema fluid clearance, one condition that it is preserved [24, 25]. To our knowledge, the effect of IH on active alveolar sodium transport has not been studied. Of note, alveolar sodium transport by alveolar epithelial cells was previously shown to be inhibited by acute and chronic hypoxia and by oxidative stress [26–28], two elements associated with IH challenge.

Oxidative stress is considered as a major pathogenic feature in IH [10], but it is also induced by BLM [29]. Here, we observed at day 4 a marked increase in 3-nitrotyrosine immunostaining as well as a trend (not significant) to an increase in 8-OH-DG levels in lung tissues from mice treated with BLM and exposed to either IA or IH. By using these techniques, we were not able to detect any significant effect of exposure to IH per se, inasmuch the Saline-IH group was not different from the Saline-IA group. These results strongly suggest that oxidative stress is induced in BLM-treated mouse lungs, with no obvious difference between mice exposed to IA and mice exposed to IH. However, quantification of antioxidant enzyme protein expression in lung homogenates revealed some differences between BLM-IA and BLM-IH groups. Namely, expression levels of SOD2 and GPX were significantly more decreased in the BLM-IH group than in the BLM-IA group (−61% versus −35%, respectively, for SOD2 and −80% versus −50%, respectively, for GPX). Although we did not measure SOD2 and GPX activities, the important decrease in protein expression levels of these enzymes suggested that antioxidant defense mechanisms might be particularly reduced in the BLM-IH group. Indeed, it was previously reported that IH decreased SOD2 activity and increased oxidative stress in lung epithelial cell line H441 cells [30]. Concerning prooxidant enzymes, our data show that lung MPO concentration was increased at day 4 in both groups treated with BLM (BLM-IA and BLM-IH), consistent with PMN infiltration of lung tissues [23]. However, whereas MPO concentration went back to normal at day 8 (corresponding to the end of the inflammatory phase of lung BLM injury) in the BLM-IA group, it remained significantly elevated in the BLM-IH group, suggesting an imbalance between lung antioxidant and prooxidant enzymes in this condition. Of note, we also evaluated mRNA and protein levels of NADPH-oxidases (NOX2 and NOX4) since these prooxidant enzymes are induced by IH in some cell types [11], but we found no significant modification under our experimental conditions (data not shown). This does not exclude however that NOX2 or NOX4 expression might vary under IH condition in some specific cell types (for instance fibroblasts or alveolar epithelial cells), but analyses performed on whole lung homogenates did not allow us to detect such cell-type specific events.

Increased apoptosis of ATII cells is a deleterious feature in IPF that compromises normal alveolar epithelium repair after injury and may therefore promote lung fibrosis [5]. Here, TUNEL staining revealed that the rate of apoptotic lung cells was markedly and specifically increased in the BLM-IH group. Apoptosis was detected both in ATII cells and immune cells. Consistent with this finding, lung expression levels of cleaved PARP protein, a marker of early apoptosis, were also significantly and specifically increased in this condition. These results are in line with previous works showing that IH can induce apoptosis in various cell types such as neurons [31] or pancreatic *β* cells [32]. Our *in vivo* model did not allow us to analyze the pro- and antiapoptotic pathways potentially involved in ATII cell apoptosis, and additional experiments *in vitro* in cultured primary ATII cells are clearly needed to study the cellular mechanisms of IH-induced apoptosis. Based on previous studies, it can be hypothesized that both activation of the hypoxia-inducible factor 1 *α* pathway (and its proapoptotic targets) and accumulation of reactive oxygen species could be involved in ATII cell apoptosis [33–35].

One important question is whether exposure to IH would worsen lung fibrosis and animal mortality in our experimental model. Our results based on lung histological examination, the time-course of extracellular matrix protein mRNA transcripts, and quantification of lung collagen provide evidence that pulmonary fibrosis at day 21 was more severe in mice treated with BLM and IH as compared with those treated by BLM and IA. Indeed, animal mortality observed during the fibrotic phase (from day 8 to day 21) was almost doubled in the BLM-IH group as compared with the BLM-IA group, and postmortem analysis of the lungs suggests that the cause of death was mostly respiratory deficiency. Our data concerning lung fibrosis are in line with those from Braun et al. [36], recently obtained in rats treated with BLM or saline on day 0 and exposed to chronic IH (nadir FIO_2_ 10%, 30 cycles/h, 10 h/day) or to normoxia from day 5 to day 35. The latter study showed that a 30-day exposure to IH increased lung NF-*κ*B activity, worsened pulmonary fibrosis (as assessed by lung hydroxyproline content), and impaired lung compliance in rats treated with BLM. The authors observed a significant increase in mRNA expression of the profibrotic factor CTGF in rat lungs treated by BLM and IH (as compared with mice treated with BLM and IA) which could contribute to worsen fibrosis, but this was not the case in our experiments in mice, maybe because of species differences. In their study, Braun et al. did not specifically evaluate the severity of pulmonary inflammation during the first week following BLM administration. Indeed, contrary to our findings, the authors did not mention any increase in the mortality rate of rats exposed to BLM and IH. This discrepancy may be due to the fact that the IH regimen they used was less severe than that in our experiments (e.g., FIO_2_ nadir 10% versus 6% in the present study). However, whatever the species and IH regimen differences between the two studies, they both strongly support the deleterious role of chronic IH in the development of BLM-induced lung fibrosis. Further *in vitro* experiments using specific lung cell types (ATII cells, fibroblasts) should be undertaken to decipher the mechanism(s) leading to the worsening of pulmonary fibrosis by chronic IH.

## 5. Conclusion

This animal study provides strong evidence that chronic IH worsens lung inflammation, lung fibrosis, and mortality in BLM-induced lung injury in mice. Our findings raise the question of the worsening impact of severe OSA in IPF patients. This represents an important issue in the clinical management of IPF for which medical therapies show little efficacy, since OSA can be efficiently treated with continuous positive airway pressure (CPAP) therapy. Prospective controlled trials evaluating the influence of OSA on the course of IPF and the potential beneficial effect of CPAP therapy in IPF patients are urgently needed to address these issues.

## Figures and Tables

**Table 1:** QPCR primers list.

Gene	Forward primer (5′-3′)	Reverse primer (5′-3)	PCR product size (bp)
*B2m*	GTGACCCTGGTCTTTCTGGT	GTATGTTCGGCTTCCCATTC	115
*Col1a1*	GTGGTGACAAGGGTGAGACA	GAGAACCAGGAGAACCAGGA	99
*Col3a1*	TACACCTGCTCCTGTGCTTC	CATTCCTCCCACTCCAGACT	226
*Fn1*	TGGTGGCCACTAAATACGAA	GGAGGGCTAACATTCTCCAG	103
*Tgfb1*	ACTGATACGCCTGAGTGGCT	CCCTGTATTCCGTCTCCTTG	80
*Ctgf*	GAGTGTGCACTGCCAAAGAT	GGCAAGTGCATTGGTATTTG	102
